# Pavlovian reward learning underlies value driven attentional capture

**DOI:** 10.3758/s13414-016-1241-1

**Published:** 2016-11-30

**Authors:** Berno Bucker, Jan Theeuwes

**Affiliations:** Department of Experimental and Applied Psychology, Vrije Universiteit Amsterdam, Van der Boechorststraat 1, 1081 BT Amsterdam, The Netherlands

**Keywords:** Visual attention, Associative reward learning, Pavlovian conditioning, Attentional capture

## Abstract

Recent evidence shows that distractors that signal high compared to low reward availability elicit stronger attentional capture, even when this is detrimental for task-performance. This suggests that simply correlating stimuli with reward administration, rather than their instrumental relationship with obtaining reward, produces value-driven attentional capture. However, in previous studies, reward delivery was never response independent, as only correct responses were rewarded, nor was it completely task-irrelevant, as the distractor signaled the magnitude of reward that could be earned on that trial. In two experiments, we ensured that associative reward learning was completely response independent by letting participants perform a task at fixation, while high and low rewards were automatically administered following the presentation of task-irrelevant colored stimuli in the periphery (Experiment 1) or at fixation (Experiment 2). In a following non-reward test phase, using the additional singleton paradigm, the previously reward signaling stimuli were presented as distractors to assess truly task-irrelevant value driven attentional capture. The results showed that high compared to low reward-value associated distractors impaired performance, and thus captured attention more strongly. This suggests that genuine Pavlovian conditioning of stimulus-reward contingencies is sufficient to obtain value-driven attentional capture. Furthermore, value-driven attentional capture can occur following associative reward learning of temporally and spatially task-irrelevant distractors that signal the magnitude of available reward (Experiment 1), and is independent of training spatial shifts of attention towards the reward signaling stimuli (Experiment 2). This confirms and strengthens the idea that Pavlovian reward learning underlies value driven attentional capture.

## Introduction

Visual selective attention is crucial for efficient everyday behavior, by enhancing the representation of stimuli that are relevant and suppressing the representation of stimuli that are potentially distracting. For example, when walking on the sidewalk, you attend fellow pedestrians and traffic signs while ignoring bypassing cars and irrelevant billboards. However, if there is a colored money bill lying on the pavement, it is likely to grab your attention. The probability that a certain stimulus is selected amongst competing stimuli is partially dependent on its perceptual features (e.g., luminance or color-contrast), and the control settings of an observer to attend to specific features or objects. Classically, the automatically stimulus-driven factors are referred to as bottom-up, and the voluntarily goal-driven factors are referred to as top-down (for reviews see Corbetta & Shulman 2002; Desimone & Duncan, 1995; Itti & Koch, 2001; Theeuwes, 2010).

However, there is more to the money bill lying on the pavement, as the learned value that the bill has acquired over years of life experience makes it likely that it captures your attention. This happens, although money on the pavement does not “stick out” per se in a bottom up fashion, and despite that we are not constantly searching for bills in a top down manner when we are walking on the sidewalk. A recent body of literature has shown that learned reward associations can evoke attentional biases that cannot be explained in terms of bottom-up and top-down factors (for reviews see Anderson, 2013, 2015; Awh, Belopolsky, and Theeuwes 2012; Chelazzi, Perlato, Santandrea, & Della Libera, 2013; Chun, Golomb, & Turk-Browne, 2011; Kristjánsson & Campana 2010, Le Pelley, Mitchell, Beesley, George, & Wills, 2016). Awh et al. (2012) argued that the attentional priority map should be extended beyond the integration of bottom-up and top-down factors, and include ‘selection history’ as a third factor driving visual selective attention. Selection history includes attentional effects due to previous selection (i.e., priming) and reward learning, and comprises that attentional selection is influenced by the significance that certain stimuli have gained over time through experience. This suggests that the deployment of attention is flexibly adapted depending on the context to maximize behavioral outcomes. As rewards in the environment are crucial for guiding optimal behavior, it is not surprising that reward signaling stimuli have a significant impact on the allocation of selective attention.

In experimental settings, it is well known that the prospect of reward can increase the motivation and effort to deliver optimal performance, thereby recruiting cognitive control processes that allow, amongst other effects, for more efficient deployment of attention (for reviews see Botvinick & Braver, 2015; Pessoa, 2009; Pessoa & Engelmann, 2010). For example, when reward is signaled at the start of a trial or block, performance is enhanced when relatively high reward can be obtained, usually characterized by a reduction in reaction time and/or improved accuracy (e.g., Bucker & Theeuwes, 2014; Sawaki, Luck, & Raymond, 2015; Small et al., 2005). Furthermore, when the delivery of relatively high reward is directly coupled to a stimulus or stimulus feature, that stimulus (feature) enjoys increased attentional priority, thereby eliciting improved behavioral performance (e.g., Bucker & Theeuwes, 2016; Kiss, Driver, & Eimer, 2009; Krebs, Boehler, Egner, & Woldorff, 2011; Munneke, Hoppenbrouwers, & Theeuwes, 2015). Hence, when reward is signaled in advance, or is congruent with the current task-demands (e.g., coupled to the spatial location or a visual feature of the target), it allows for optimal preparation and strategic adjustment of attentional processes in order to achieve more efficient goal-directed behavior. This intuitively makes sense, as the best strategy to maximize reward income under these circumstances is to integrate and prioritize the reward signal in the process of attentional selection.

However, it has also been shown that learned reward associations can trigger attentional prioritization that goes against the goal-directed state of the observer, and can be detrimental for task performance (e.g., Anderson, Laurent, & Yantis 2011a, 2011b; Della Libera & Chelazzi, 2006, 2009; Failing & Theeuwes, 2014, Hickey, Chelazzi, & Theeuwes, 2010a, 2010b). For example, Hickey et al. (2010a, 2010b) showed that, when high and low rewards were distributed at random, attention was biased towards the color that was high rewarded on the previous trial, not only when the target was presented in the high reward color on the current trial, but also when the distractor was presented in the high reward color on the current trial. This suggests that high reward stimuli automatically capture attention, and elicit an attentional bias towards features associated with high reward, even when selection of those features runs against the current goals of the observer and is detrimental for task performance and monetary payout.

In addition, several studies have shown that reward associated stimuli are prioritized, even when reward features become completely task-irrelevant in a context where the actual rewards are no longer delivered (e.g., Anderson et al. 2011a, 2011b; Bucker, Silvis, Donk, & Theeuwes, 2015; Della Libera & Chelazzi, 2006; 2009; Failing & Theeuwes, 2014; MacLean, Diaz, & Giesbrecht, 2016). Typically, these studies make use of (1) an initial training phase in which target features are associated with high and low reward-value, and (2) a test phase in which the previously reward signaling target features become distractor features and rewards are no longer delivered. For example, in Anderson et al. (2011a, 2011b), during an initial training phase, participants had to search for a red or green circle amongst multiple colored circles, and discriminate the orientation of a line segment within the target circle. For successful selection of the colored target circle, followed by a correct manual response, participants earned either a high or low reward depending on the color-reward contingencies (e.g., red gives a high chance on high reward and a low chance on low reward, and green gives a high chance on low reward and a low chance on high reward). Then, during the following test phase, participants had to search for an odd-shaped target amongst several distractors that had the same shape and report the orientation of a line segment within the odd-shaped target (i.e., the additional singleton paradigm, Theeuwes, 1992). Rewards could no longer be earned, but one of the distractors could occasionally appear in the previously high or low reward signaling color. Even though participants were instructed to ignore any color information, the distractor slowed search significantly when it was presented in the color previously associated with the high reward compared to the color previously associated with the low reward (Anderson et al., 2011a) or compared to when none of the reward colors were present (Anderson et al., 2011b). This suggests, that so-called value driven attentional capture (see Anderson, 2013) occurs even when the high reward-value associated stimulus (feature) is task-irrelevant, physically non-salient and no longer predictive for reward delivery.

Recently it has been argued that value-driven attentional capture as described above could be the result of stimulus–response learning rather than the mere statistical regularities between the presence of a stimulus and reward delivery (see Le Pelley et al., 2016). By repeatedly reinforcing attentional orienting towards high reward features in a training phase, this learned attentional response becomes a selection bias that persists during a following test phase, even if shifting attention towards the same stimulus features is now detrimental for task performance. In other words, it is possible that the observed attentional capture by the previously reward signaling stimuli during the test phase is a carryover of attentional orienting that was initially trained during the training phase. This would suggest that instrumental rather than Pavlovian conditioning underlies the effects of value driven attentional capture. To investigate the associative learning mechanism underlying value driven attentional capture, Le Pelley and colleagues (Le Pelley, Pearson, Griffiths, & Beesley, 2015) designed a variation of the additional singleton paradigm in which high or low rewards were delivered for fast and correct target responses depending on the color of a task-irrelevant distractor. Crucially, attentional orienting towards the distractor stimulus needed to be suppressed in order to give a correct response in time and receive that trial’s reward. In fact, orienting towards the reward signaling distractor item resulted in the omission of reward. The authors reasoned that if instrumental learning underlies value-driven attentional capture, behavior should be more efficient on high compared to low reward trials as suppressing attentional orienting towards the reward item is reinforced, making it easier to perform the target discrimination. However, if Pavlovian learning underlies value-driven attentional capture, behavior should be less efficient on high compared to low reward trials, as the reward signaling distractor automatically captures attention, making it more difficult to perform the target discrimination. The results of Le Pelley et al. (2015) showed the high compared to low reward signaling distractors captured attention more strongly, and similar findings have been reported for other behavioral (Mine & Saiki, 2015) and oculomotor tasks (Bucker, Belopolsky, & Theeuwes, 2015; Failing, Nissens, Pearson, Le Pelley, & Theeuwes, 2015; McCoy & Theeuwes 2016; Pearson, Donkin, Tran, Most, Le Pelley, 2015). This suggests that Pavlovian rather than instrumental reward learning underlies the effects of value-driven attentional capture.

However, in all previous studies, reward administration was response dependent, as only fast and correct responses were followed by actual reward delivery. Furthermore, the stimulus that signaled the availability of reward was presented within the stimulus display, and the only way for participants to find out how much reward they could earn on any given trial was to attend the color of the reward signaling distractor. In all previous studies, except for Mine and Saiki (2015), which utilized a design with a training and test phase, value-driven attentional capture was assessed while the reward associated distractor was present to predict the reward outcome of a trial. This implies that the reward associated distractor was never completely task-irrelevant as it conveyed the informational value of the reward magnitude that was available (i.e., signaling how much reward could be earned on a particular trial). To rule out any influence of task-relevance, we therefore separated the associative reward learning phase from the test phase in which value-driven attentional capture was assessed in the present study. Although Mine and Saiki (2015) also used a separate training phase to associate distractor stimuli with differential reward-value, the distractor stimuli always appeared in the target stimulus display at the time a response was required. Furthermore, only correct responses were rewarded, making associative reward learning response dependent. Thus, in all previous studies trying to uncover the learning mechanism underlying value-driven attentional capture associative reward learning was never truly Pavlovian, and the reward associated distractors were never completely separated from the task at hand. In the present study, we aimed to specifically address whether the mere co-occurrence of a stimulus and reward administration, without any instrumental relationship in obtaining reward, could lead to value-driven attentional capture.

In order to examine whether genuine Pavlovian reward learning underlies value-driven attentional capture, we conducted two experiments. In both experiments, a Pavlovian reward conditioning phase was separated from a non-reward test phase using the additional singleton paradigm. This ensured that there was no motivation whatsoever to attend towards the reward-value associated stimuli in order to check how much reward could be earned on any given trial. Crucially, during associative reward learning in the reward conditioning phase, reward administration was also completely task- and response-independent. Participants fixated the central fixation cross and had to respond to a 45° angular degrees rotation of this fixation cross. While fixating the central fixation cross, circles or diamonds (one of which was presented in color) appeared in the periphery and were, independent of the response of the participant, followed either by high or low reward administration. The reward signaling distractors and reward feedback were never presented simultaneously with a change of fixation, such that associative reward learning was separated from the fixation change task in time and that the delivery of reward could not coincidentally be attributed to any key press or performance on the assigned task. In Experiment 1, stimuli following high and low reward administration were presented in the periphery, such that the location where reward learning occurred was spatially separate from the task at fixation. In Experiment 2, the stimulus display contained only one colored stimulus, which was presented at the focus of attention at fixation, to prevent spatial orienting towards the reward signaling stimuli.

The same non-reward test phase followed the reward conditioning phase in both experiments, to examine value-driven attentional capture. During the non-reward test phase, rewards were no longer administered, and participants had to perform a discrimination task on a bar stimulus that was presented in a gray shape singleton amongst several non-target shapes. Crucially, one of the distractor shapes was sometimes presented in the high or low reward-value associated color. We expected that the colored singletons that previously signaled reward would attract attention and slow search relative to trials without a colored singleton distractor. Furthermore, if Pavlovian learning underlies value driven attentional capture, performance should be impaired on trials in which a high compared to low reward-value associated distractor stimulus was present. However, if associative reward learning involves an instrumental aspect and is dependent on the association of a response with reward delivery, we expect to find no performance differences, between high and low reward-value distractor trials in the non-reward test phase. Altogether, these experiments should give clear insight in whether associative reward learning through pure Pavlovian conditioning elicits value-driven attentional capture.

## Experiment 1

**Fig. 1:**
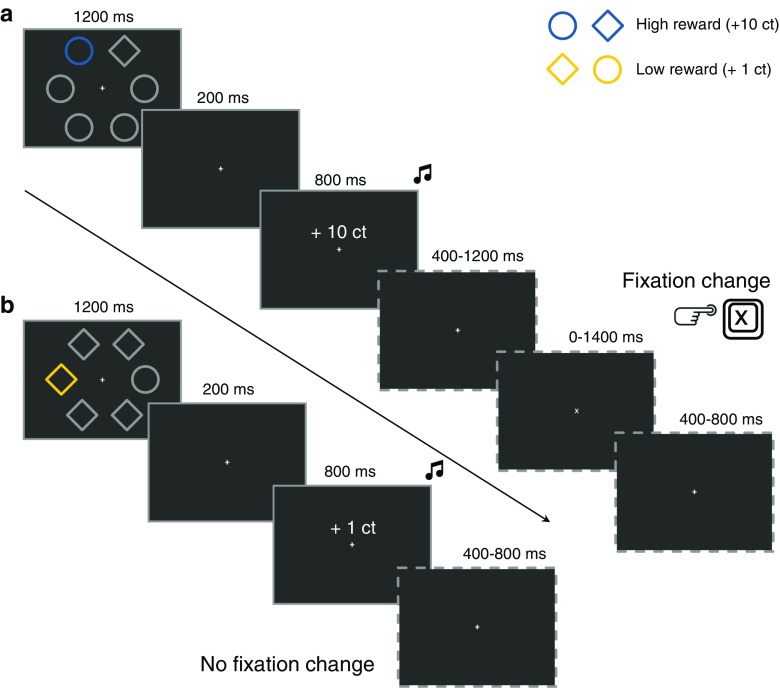
**a**,**b** Schematic representation of the trial sequence and timing of the reward conditioning phase in Experiment 1. **a** high reward stimulus display (*blue circle*) and high reward feedback display indicating a 10 cent win, followed by a fixation change trial (fixation changed from “+” to “x”) on which participants had to press the keyboard “X” button within 1400 ms. **b** A low reward stimulus display (*yellow diamond*) and low reward feedback display indicating a 1 cent win, followed by a no fixation change trial on which no response was required

**Fig. 2:**
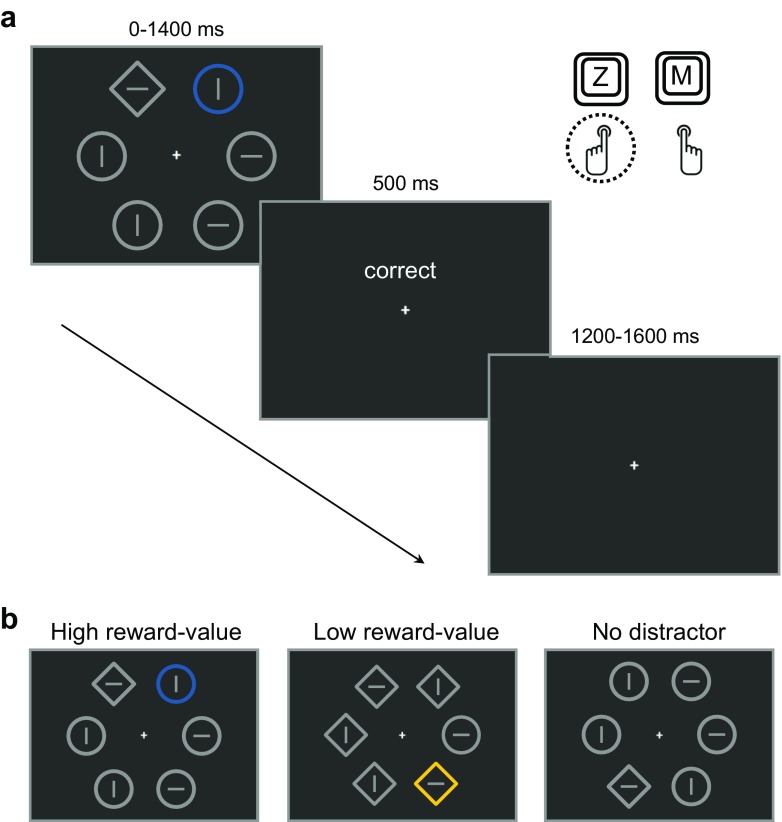
**a**,**b** Schematic representation of the trial sequence and timing of the non-reward test phase. **a** The stimulus display of the additional singleton paradigm with a high reward-value color singleton distractor (*blue circle*), and a *diamond shape* singleton target containing a *horizontal line element* for which a “Z” key press within 1400 ms resulted in the correct feedback screen. **b** Overview of the three different distractor type conditions

During the reward conditioning phase, participants performed a task at fixation, while completely task-irrelevant stimuli were presented in the periphery (see Fig. 1). One of these stimuli was presented in a color that could be associated with the delivery of either a high or a low reward. Crucially, the stimuli in the periphery and reward administration were completely task irrelevant and not associated with any response. Thus, through Pavlovian reward learning, we associated specifically colored stimuli with either high or low reward-value. In the non-reward test phase, rewards were no longer administered, and participants performed a discrimination task on a bar stimulus presented in a gray shape singleton amongst several non-target shapes (see Fig. 2). Crucially, one of the distractor shapes was sometimes presented in one of the previously reward signaling colors. We examined value-driven attentional capture by comparing performance between trials in which a high and low reward-value associated distractor was present.

## Methods

### Participants

For Experiment 1, a group of 24 participants [7 males, 20–31 years of age, mean = 23.5 years, standard deviation (SD) = 2.7 years] was tested at the Vrije Universiteit Amsterdam. All participants reported having normal or corrected-to-normal vision, and gave written informed consent before participation. Participants earned € 5.28 reward during the reward conditioning phase, and were paid at a rate of € 8.00 per hour to compensate for participation. As the experiment lasted approximately 40 min, participants earned around € 10.00 in total. All research was approved by the Vrije Universiteit Faculty of Psychology ethics board, and was conducted according to the principles of the Declaration of Helsinki.

### Apparatus

All participants were tested in a sound-attenuated, dimly-lit room, with their head resting on a chinrest at a viewing distance of 75 cm. A computer with a 3.2 GHz Intel Core processor running OpenSesame (Mathôt, Schreij, & Theeuwes, 2012) generated the stimuli on a 22-inch screen (resolution 1680 × 1050, refreshing at 120 Hz). The necessary response data were acquired through the standard keyboard and all auditory stimuli were presented through headphones.

## Stimuli

### Reward conditioning phase

Throughout the reward conditioning phase, a white (CIE: *x* = .255, *y* = .437, 67.11 cd/m^2^) fixation cross (.30° x .30°) was presented on a black (<1 cd/m^2^) background at the center of the screen (see Fig. 1). On fixation-change trials (25 % of the trials) the fixation cross (“+”) rotated 45° angular degrees (“x”) for a brief period, but the fixation cross never disappeared from the screen. The reward stimulus display was based on the additional singleton paradigm display (Theeuwes, 1992) but did not contain target or distractor line elements. Accordingly, six shapes, randomly one diamond (2.95° x 2.95°) amongst five circles (*r* = 1.45°) or one circle amongst five diamonds, were presented at equal distances on an imaginary circle (*r* = 5.90°). Five stimuli were presented in gray (CIE: *x* = .308, *y* = .335, 22.1 cd/m^2^), and one color singleton was presented in either blue (CIE: *x* = .190, *y* = .197, 22.5 cd/m^2^) or yellow (CIE: *x* = .412, *y* = .513, 22.6 cd/m^2^). The shape singleton was never presented in color. The locations of the shape and color singleton were assigned randomly on each trial. The reward feedback display contained the written text “+1 ct” or “+10 ct” (~.5° x 3.0°) presented in white (CIE: *x* = .255, *y* = .437, 67.11 cd/m^2^), 0.50° visual degrees above the fixation cross at the center of the screen. Simultaneously with the visually presented feedback, the sound of one or more dropping coins was played in low and high reward trials, respectively.

The reward stimulus display was presented for 1200 ms followed by a 200-ms interval during which the fixation cross was shown. Then the reward feedback display was presented for 800 ms. On no fixation change trials, the fixation cross was presented for a random inter-trial interval of 400–800 ms after which the next reward stimulus display was shown. On fixation change trials the reward feedback display was followed by a random 400–1200 ms interval, after which the fixation cross rotated 45° angular degrees. When this change occurred participants had to press the “X” button on the keyboard within a 1400 ms response window. Immediately following the button press, or after 1400 ms, the fixation cross rotated back to normal and the standard 400–800 ms inter-trial interval was followed by the presentation of the next reward stimulus display.

### Non-reward test phase

For the non-reward test phase, we utilized the additional singleton task (Theeuwes, 1992). Accordingly, six shapes, randomly one diamond (2.95° x 2.95°) amongst five circles (*r* = 1.45°) or one circle amongst five diamonds, were presented at equal distances on an imaginary circle (*r* = 5.90°) (see Fig. 2). All stimuli were presented in gray (CIE: *x* = .308, *y* = .335, 22.1 cd/m^2^), or all were presented in gray except for the color singleton, which was presented in either blue (CIE: *x* = .190, *y* = .197, 22.5 cd/m^2^) or yellow (CIE: x = .412, y = .513, 22.6 cd/m^2^). The shape singleton was never colored and always presented in gray. All shapes contained a gray (CIE: *x* = .308, *y* = .335, 22.1 cd/m^2^) horizontal or vertical line element (0.50° x 3 pixels). At all times a white (CIE: *x* = .255, *y* = .437; 67.11 cd/m^2^) fixation cross (.30° x .30°) was presented at the center of the screen. Feedback consisted of the written words (~.5° x 2.0°), “correct”, “incorrect” or “too slow” presented in white (CIE: *x* = .255, *y* = .437; 67.11 cd/m^2^). Visual feedback for incorrect and too slow response trials was accompanied by a pure tone (sine wave) of 400 Hz and 800 Hz, respectively.

The stimulus display was presented for a maximum period of 1400 ms, and disappeared as soon as a response was given. The stimulus display was directly followed by the visual feedback for 500 ms, occasionally accompanied by the auditory feedback that was played for 200 ms. All trials were separated by a random 1200–1600 ms inter-trial interval.

### Procedure and design

Participants were instructed to remain fixated at the central fixation cross at all times, and to respond as quickly and accurately as possible. In the reward conditioning phase, the task for participants was to press the “X” keyboard button as soon as the fixation cross rotated 45° angular degrees. They were instructed that other stimuli would appear in the periphery but that these were not related to their task and could be ignored. Furthermore, they were told that they would automatically receive rewards while doing the fixation task, and that reward administration was independent of the assigned task and corresponding response. The reward conditioning phase consisted of three separate blocks with 32 trials each. Within each block the high and low reward stimulus display were both shown 16 times in random order. The color-reward contingencies (i.e., blue and yellow for high and low rewards) were counterbalanced across participants. The fixation cross changed eight times per block (25 % of the time) randomly in between trials. In between blocks of the reward conditioning phase, participants were shown on how many occasions they missed the fixation change and their total reward received. In total, 96 reward displays were shown and 24 fixation changes occurred during the reward conditioning phase.

At the start of the non-reward test phase, participants were explicitly told that rewards were no longer delivered. Furthermore, they were told that the fixation change task was finished, but that fixation needed to be maintained. The task in the non-reward test phase was to discriminate the orientation of the target line within the odd-shaped figure (i.e., the shape singleton), and to press the “Z” or “M” button as fast and accurate as possible for horizontal or vertical line elements respectively. The test phase consisted of eight blocks with 36 trials each. Within each block there were 12 high reward-value distractor trials, 12 low reward-value distractor trials and 12 no distractor trials. The shape singleton contained a horizontal or vertical target line equally often within each block. In between blocks, participants were informed about their mean response time and mean accuracy in that particular block and their overall accuracy. In total, 288 trials were completed in the non-reward test phase. Including breaks in between blocks, all participants were able to finish the experiment within approximately 40 min.

### Statistical analyses

Accuracy was calculated as the percentage correct of all trials. All correct responses that were made within the 1400 ms response window were included in the accuracy analysis. For the reaction time analysis, only correct trials were analyzed. Furthermore, trials on which reaction time deviated more than 2.5 SD from the participant’s individual overall mean correct reaction time (2.0 % of the correct reaction time data), were excluded from the analysis.

In order to investigate possible speed accuracy tradeoffs on an individual level, and in order to compare distractor condition differences in speed and/or accuracy between Experiments 1 and 2, we calculated the inverse efficiency score (IES), first proposed by Townsend and Ashby (1978, see also Townsend and Ashby 1983). IES (reaction time / proportion of correct responses) reflects the average energy consumed by the system over time, and can properly be used as a measure of performance in addition to reaction time and accuracy (Bruyer & Brysbaert, 2011). In order to examine the effects of reward on attention, IES has been used before in Shomstein & Johnson (2013).

To investigate whether Pavlovian reward conditioning could lead to value-driven attentional capture, a repeated measures ANOVA with distractor type (high reward-value/low reward-value/no distractor) as factor was performed on reaction time, accuracy and IES. The effect of distractor presence (high and low reward-value distractor trials compared to no distractor trials) and the effect of value driven attentional capture (high compared to low reward-value distractor trials) were investigated using two-tailed paired samples *t*-tests.

### Exclusions

We excluded participants from the analyses if performance in one or more conditions of the non-reward test phase was at or below chance level. The cumulative binomial distribution indicated that performance significantly (*P* < .041) deviated from chance (50 %) if participants responded correctly on 56 or more trials out of a total of 96 trials per condition. Thus, participants who scored below 58 % correct in one or more conditions were excluded from the analysis. In Experiment 1, all participants scored above this criterion.

## Results

### Reward conditioning

Out of 24 subjects, one subject missed the fixation change once and one subject showed a false alarm in the reward conditioning phase. As reward was independent of the fixation task and any response, all participants earned € 5.28 extra reward.

### Value-driven attentional capture

**Fig. 3:**
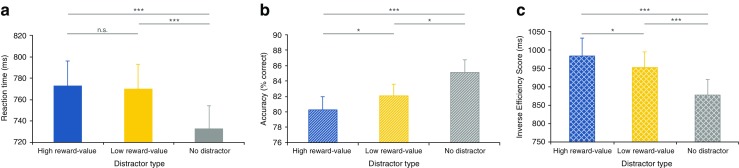
**a**–**c** Results Experiment 1. **a** Mean reaction time per condition. **b** Mean accuracy per condition. **c** Mean inverse efficiency score (reaction time/percentage correct responses) per condition. Note that accuracy is significantly lower and that the inverse efficiency score is significantly higher (and performance thus worse) in the high compared to the low reward-value distractor condition. *Error bars* indicate Standard errors (SE) of the means

To investigate whether associative reward learning through Pavlovian conditioning elicited value-driven attentional capture in the non-reward test phase, we compared mean reaction time, accuracy and IES between the high reward-value, low reward-value and no distractor condition in the test phase (see Fig. 3).

### Reaction time

A repeated measures ANOVA on mean reaction time with distractor type (high reward-value/low reward-value/no distractor) as factor showed a significant main effect, *F*(2, 46) = 33.823, *P* < .001, η_p_^2^ = .595. However, a planned paired samples two-tailed *t*-test showed that reaction times the high reward-value (mean = 772 ms) and the low reward-value (mean = 768 ms) distractor condition, although numerically, did not significantly differ (*t* < 1). Subsequent planned paired samples two-tailed *t*-tests showed that reaction times were significantly shorter in the no distractor condition (mean = 733), compared to both the high *t*(23) = 6.455, SE = 5.992, *P* < .001, η_p_^2^ = .644, and low, *t*(23) = 7.251, SE = 4.897, *P* < .001, η_p_^2^ = .696, reward-value distractor condition. These results show that participants significantly slowed their responses when one of the reward-value distractors was present compared to the condition in which no distractor was present, although no significant slowing was observed when high compared to low reward-value distractors were present. However, participants performed significantly less accurately in the high compared to the low reward-value distractor condition.

### Accuracy

A repeated measures ANOVA on mean accuracy with distractor type (high reward-value/low reward-value/no distractor) as factor showed a significant main effect, *F*(2, 46) = 11.565, *P* < .001, η_p_^2^ = .335. Crucially, as already indicated, a subsequent paired samples two-tailed *t*-tests showed that accuracy was significantly worse in the high (mean = 80 %) compared to low (mean = 82 %) reward-value distractor condition, *t*(23) = 2.172, *SE* = .839, *P* = .040, η_p_^2^ = .170 . Furthermore, performance was better in the no distractor condition (mean = 85 %) compared to both the high, *t*(23) = 4.563, SE = 1.065, *P* < .001, η_p_^2^ = .475, and low reward-value distractor condition, *t*(23) = 2.676, SE = 1.135, *P* = .013, η_p_^2^ = .237.

### Efficiency

In order to confirm a genuine performance deficit in the high compared to the low reward-value distractor condition, we made use of the IES. A repeated measures ANOVA on IES with distractor type (high reward-value/low reward-value/no distractor) as factor showed a significant main effect, *F*(2, 46) = 26.760, *P* < .001, η_p_^2^ = .528. Crucially IES was higher in the high (mean = 984 ms) compared to low (mean = 953 ms) reward-value distractor condition, *t*(23) = 2.259, SE = 13.883, *p* = .034, η_p_^2^ = .182, confirming that search performance was significantly less efficient when a high compared to a low reward-value distractor was present. Furthermore, paired samples two-tailed *t*-tests showed that search performance was more efficient in the no distractor condition (mean = 878 ms) compared to both the high, *t*(23) = 7.345, *SE* = 14.451, *P* < .001, η_p_^2^ = .701, and low, *t*(23) = 4.592, *SE* = 16.282, *P* < .001, η_p_^2^ = .478, reward-value distractor condition.

## Discussion

Altogether, the results of Experiment 1 show that participants respond faster, more accurately and more efficiently on trials in which no distractor is presented compared to trials in which a reward-value-associated distractor is present. Crucially, performance was significantly impaired when high compared to low reward-value associated distractors were presented, illustrated by a significant decline in accuracy and search efficiency. These results suggests that associative reward learning of the stimulus-reward contingencies can occur completely temporally and spatially independent of the assigned task, such that value-driven attentional capture is observed in a following non-reward test phase. This confirms and strengthens the idea that Pavlovian reward learning underlies value-driven attentional capture.

## Experiment 2

As the reward signaling color singletons were presented in the periphery in the reward conditioning phase of Experiment 1, it is possible that learning the color-reward contingencies involved orienting of attention towards them. Even though reward administration was completely task- and response-independent, it is thus possible that associative reward learning in Experiment 1 still involved an instrumental aspect, as attention might have been captured by the color singleton in the periphery. Indeed, it has been shown that color singletons can capture attention in an automatic fashion, even when it is not relevant for the task (Theeuwes, 1992, 2010). If such automatic instrumental conditioning took place during reward conditioning, it is possible that training this instrumental response was responsible for the value driven attentional capture effect that was observed in the non-reward test phase. This is of specific importance, as the shift of attention towards the reward signaling color singletons in the reward conditioning phase matches the attentional response towards the reward-value associated distractors in the non-reward test phase that constitutes value-driven attentional capture.

In order to show that true Pavlovian reward learning underlies value-driven attentional capture, we ensured that no automatic shifts of attention towards the reward signaling color singleton could occur during associative reward learning in Experiment 2. Therefore, we presented a single colored stimulus at the focus of attention at fixation, such that there was no need for a spatial shift of attention towards the periphery, hereby eliminating every instrumental aspect of associative reward learning.

### Method

In general, the method of Experiment 2 was highly similar to that of Experiment 1.

### Participants

For Experiment 2, a new group of 26 participants (11 males, 20–34 years of age, mean = 25.0 years, SD = 3.4 years) was tested at the Vrije Universiteit Amsterdam. All participants reported having normal or corrected-to-normal vision, and gave written informed consent before participation. Participants earned € 5.28 reward during the reward conditioning phase, and were paid at a rate of € 8.00 per hour to compensate for participation. As the experiment lasted approximately 40 min, participants earned around € 10.00 in total. All research was approved by the Vrije Universiteit Faculty of Psychology ethics board, and was conducted according to the principles of the Declaration of Helsinki.

### Apparatus

Experiment 2 and Experiment 1 were conducted with the same equipment under the same conditions.

## Stimuli

### Reward conditioning phase

**Fig. 4:**
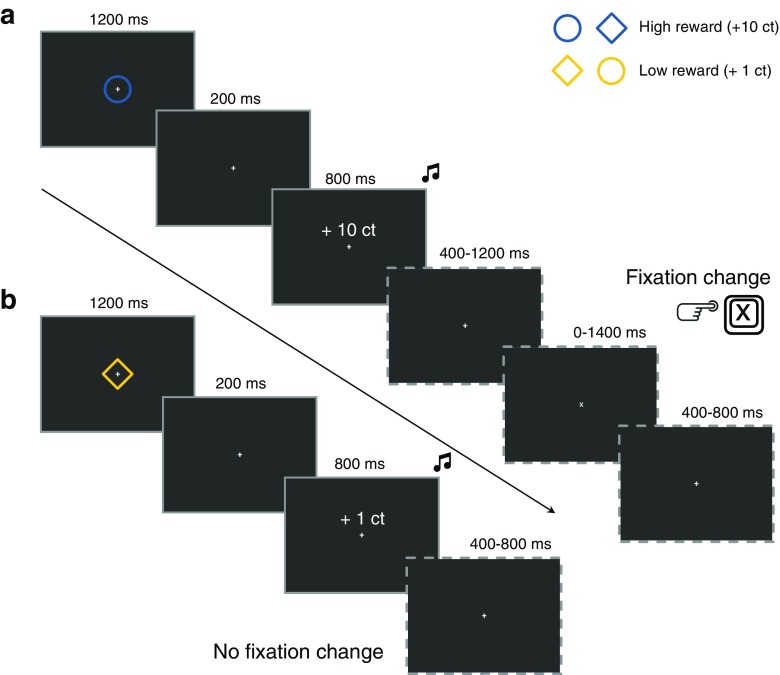
**a**,**b** Schematic representation of the trial sequence and timing of the reward conditioning phase in Experiment 2. **a** high reward stimulus display containing only one colored stimulus (*blue circle*), followed by a high reward feedback display indicating a 10 cent win, followed by a fixation change trial (fixation changed from “+” to “x”) on which participants had to press the keyboard “X” button within 1400 ms. **b** A low reward stimulus display containing only one colored stimulus (*yellow diamond*), followed by a low reward feedback display indicating a 1 cent win, followed by a no fixation change trial on which no response was required

The stimuli and timings used in the reward conditioning phase of Experiment 2 (see Fig. 4) were very similar to those used in Experiment 1. However, instead of presenting six shapes (including one color singleton) around fixation in Experiment 1, only one colored stimulus was presented at fixation, exactly in the middle of the screen, in Experiment 2. By presenting the colored stimuli in the reward conditioning phase at the focus of attention at fixation, no spatial attentional orienting towards the reward signaling stimuli occurred. As in Experiment 1, one of the colors (blue or yellow) was consistently coupled to high reward (10 cents) administration and the other color was consistently coupled to low reward (1 cent) administration.

### Non-reward test phase

The stimuli and timings used in the non-reward test phase were identical to those used in Experiment 1 (see Fig. 2).

### Statistical analyses

The data in Experiment 2 were handled identical to those in Experiment 1. In Experiment 2, due to a deviation of more than 2.5 SD from the participant’s individual overall mean correct reaction time, 2.4 % of the correct reaction time data were excluded from the reaction time analysis.

### Exclusions

As in Experiment 1, we excluded participants from the analyses if performance in one or more conditions of the non-reward test phase was at or below chance level (50 %). In Experiment 2, two participants scored at or below chance level (i.e., below 58 % correct) in one or more conditions and were therefore excluded from the analyses.

## Results

### Reward conditioning

Out of 24 subjects, one subject missed the fixation change once and one subject missed the fixation change four times out of a total of 24 fixation changes. Furthermore, one subject showed one false alarm and two other subjects showed two false alarms during the reward conditioning phase. As reward was independent of the fixation task and any response, all participants earned € 5.28 extra reward.

### Value-driven attentional capture

**Fig. 5:**
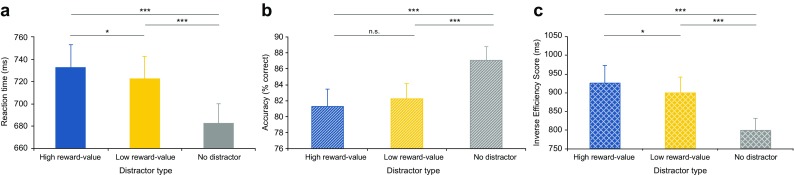
**a**–**c** Results Experiment 2. **a** Mean reaction time per condition. **b** Mean accuracy per condition. **c** Mean inverse efficiency score (reaction time/percentage correct responses) per condition. Note that reaction time is significantly slower and that the inverse efficiency score is significantly higher (and performance thus worse) in the high compared to the low reward-value distractor condition. *Error bars* indicate SE of the means

To investigate whether value-driven attentional capture following Pavlovian learning is independent of training attentional orienting towards reward signaling stimuli, we presented the reward signaling stimuli in the reward conditioning phase at fixation and compared mean reaction time, accuracy and IES between the high reward-value, low reward-value and no distractor condition in the non-reward test phase (see Fig. 5).

### Reaction time

A repeated measures ANOVA on mean reaction time with distractor type (high reward-value/low reward-value/no distractor) as factor showed a significant main effect, *F*(2, 46) = 33.949, *P* < .001, η_p_^2^ = .596. Crucially, planned paired samples two-tailed *t*-test showed that participants responded significantly slower in the high (mean = 729 ms) compared to the low (mean = 720 ms) reward-value distractor condition, *t*(23) = 2.278, SE = 3.758, *P* = .032, η_p_^2^ = .184. Subsequent planned paired samples two-tailed *t*-tests showed that reaction times were significantly shorter in the no distractor condition (mean = 682), compared to both the high *t*(23) = 6.856, SE = 6.802, *P* < .001, η_p_^2^ = .671, and low, *t*(23) = 5.465, SE = 6.967, *P* < .001, η_p_^2^ = .565, reward-value distractor condition.

### Accuracy

A repeated measures ANOVA on mean accuracy with distractor type (high reward-value/low reward-value/no distractor) as factor showed a significant main effect, *F*(2, 46) = 14.869, *P* < .001, η_p_^2^ = .393. A subsequent paired samples two-tailed *t*-tests showed that, although participants were less accurate in the high (81 %) compared to the low (82 %) reward-value distractor condition, accuracy did not differ significantly, *t*(23) = 1.440, SE = .663, *P* = .163, η_p_^2^ = .083. Further planned paired samples two-tailed *t*-tests showed that performance was better in the no distractor condition (mean = 87 %) compared to both the high, *t*(23) = 4.121, SE = 1.401, *P* < .001, η_p_^2^ = .425, and low reward-value distractor condition, *t*(23) = 3.985, SE = 1.209, *P* < .001, η_p_^2^ = .408.

### Efficiency

As in Experiment 2, in order to confirm a genuine performance deficit in the high compared to the low reward-value distractor condition, and to take possible speed-accuracy tradeoffs at an individual level into account, we calculated the IES (reaction time / proportion of correct responses). A repeated measures ANOVA on IES with distractor type (high reward-value/low reward-value/no distractor) as factor showed a significant main effect, *F*(2, 46) = 28.697, *P* < .001, η_p_^2^ = .555. Crucially, the IES was higher in the high (mean = 924 ms) compared to low (mean = 898 ms) reward-value distractor condition, *t*(23) = 2.608, SE = 10.007, *p* = .016, η_p_^2^ = .228, confirming that performance was less efficient when a high compared to a low reward-value distractor was present. Furthermore, paired samples two-tailed *t*-tests showed that search performance was more efficient in the no distractor condition (mean = 789 ms) compared to both the high, *t*(23) = 5.823, *SE* = 21.642, *P* < .001, η_p_^2^ = .596, and low, *t*(23) = 5.291, *SE* = 18.885, *p* < .001, η_p_^2^ = .549, reward-value distractor condition. These results show that search was significantly less efficient when one of the reward-value distractors was present compared to the condition in which no reward-value associated distractor was present, and, crucially, that a significant impairment in search performance was observed when high compared to low reward-value distractors were present.

## Discussion

Altogether, the results of Experiment 2 show that high and low reward-value associated distractors significantly slow search and impair search efficiency compared to a no distractor condition. Crucially, participants were significantly slower, and showed less efficient search behavior, when the high compared to the low reward-value distractor was presented in the periphery in the non-reward test phase. This suggests that a possible initial automatic shift of attention to a peripherally presented color singleton during reward learning, as might have taken place during Experiment 1, did not play a role in associative reward learning. The fact that learning or training to shift attention towards reward signaling stimuli is not required in order for observers to later show value-driven attentional capture (i.e., shifting attention towards high and low reward-value associated stimuli), emphasizes the Pavlovian nature of associative reward learning underlying value-driven attentional capture.

## General discussion

In two experiments, we examined whether true Pavlovian conditioning would be sufficient to obtain value-driven attentional capture. More specifically, we investigated whether the mere co-occurrence between the presentation of colored stimuli and the administration of high and low rewards was able to elicit value driven attentional capture in a subsequent test phase during which rewards were no longer delivered. In the reward conditioning phase, we ensured that associative reward learning was completely task- and response-independent by letting participants perform a task at fixation, while the rewards were automatically administered following the presentation of task-irrelevant colored stimuli. In Experiment 1, the reward signaling stimuli were presented in the periphery, such that the reward structure was both separated in time and in place from the assigned task during associative reward learning. In Experiment 2, the reward signaling stimuli were presented at fixation, to eliminate the possible instrumental aspect of shifting attention towards the reward signaling stimuli during associative reward learning.

The results show that performance in the non-reward test phase was impaired on trials in which a high compared to low reward-value associated distractor was presented. This suggests that associative reward learning through Pavlovian conditioning imbued the reward signaling items with value. In other words, Pavlovian learning of value signals in the reward conditioning phase elicited value-driven attentional capture in the non-reward test phase. Furthermore, the results show that performance was impaired on trials in which one of the reward-value associated distractors was present compared to trials in which no distractor was present, which is in line with previous studies showing value-driven attentional capture (Anderson et al., 2011a, 2011b) and the original findings regarding the additional singleton task demonstrating attentional capture by physically salient singletons (Theeuwes, 1992). Together, the results of these two experiments provide clear evidence that value-driven attentional capture occurs following associative reward learning through pure Pavlovian conditioning.

The findings of the present study are in line with previous studies suggesting that Pavlovian learning of stimulus-reward contingencies rather than instrumental reward learning underlies value-driven attentional capture (Le Pelley et al., 2015; Pearson et al., 2015; Failing et al., 2015). In these previous studies, reward administration was not contingent on responding to the stimuli that signaled the magnitude of the available reward. In fact, these experiments were explicitly designed in such a way that instrumental reward learning and Pavlovian reward learning would produce opposite results. Likewise, the present study was explicitly designed to examine whether pure Pavlovian conditioning during an associative reward learning phase could produce value-driven attentional capture in a non-reward test phase. However, in prior studies using a training and test phase such as in Anderson et al. (2011a, 2011b), it cannot be distinguished whether value-driven attentional capture occurred as a consequence of instrumental or Pavlovian reward learning, as the reward-value associated stimuli enjoyed increased attentional priority because of both their selection and reward history (see Awh et al., 2012). Typically, participants learned to attend and select reward associated target stimuli during a training phase, which then captured attention when they appeared as distractors in a non-reward test phase. Using such a design, associative reward learning can be due to (1) learning to attend the reward-value associated stimuli during training (i.e., selection history), or (2) the mere co-occurrence of those stimuli with reward administration (i.e., reward history). Thus, when attentional selection is congruent with the value signal, one cannot distinguish whether instrumental associative reward learning by means of a trained attentional response, or Pavlovian associative reward learning as a result of the regularities between the presentation of specific stimuli and reward delivery, underlies value-driven attentional capture. However, the present results suggest that reward history, independent of selection history, is already sufficient to elicit value-driven attentional capture. Nevertheless, future research is needed to examine whether training an attentional response that is congruent with the value signal elicits, for example, stronger capture or capture that is more resistant to extinction, compared to value-driven attentional capture that underlies pure Pavlovian conditioning.

Compared to all previous studies, the present study is unique because the reward signaling stimuli in Experiment 2 were presented at the focus of attention. By presenting the reward signaling stimuli at the focus of attention at fixation, it is unlikely that a shift of spatial attention towards them occurred, as participants were already fixated at fixation for the assigned task. By preventing any form of attentional orienting towards the reward signaling stimuli during the reward conditioning phase, we eliminated all possible instrumental aspects of associative reward learning. This was not the case in previous studies (Failing et al. 2015; Le Pelley et al., 2015; Mine & Saiki, 2015; Pearson et al., 2015), as the reward associated distractor was never presented at fixation before. In the behavioral study by Mine and Saiki (2015), the reward signaling distractors in the training phase were always presented in the periphery, leaving the option that participants covertly or overtly shifted attention towards them during reward learning. Although overt shifts of attention towards the reward signaling distractor stimuli resulted in reward omission in the oculomotor paradigms of Le Pelley et al. (2015) and Pearson et al. (2015), the reward predictive stimuli were physically salient, and therefore might have automatically captured covert attention. As the authors argue (see, Le Pelley et al., 2015), this provides circumstances under which a form of instrumental conditioning (i.e., superstitious conditioning, see Skinner, 1948) could favorably promote covert attentional shifts towards the high compared to the low reward signaling distractor in the future. If the greater likelihood of covert attentional shifts towards the high reward signaling distractor translates into making more overt shifts towards this distractor, it can account for the observed oculomotor value-driven attentional capture effect. Similarly, even though being physically non-salient, it can be argued that the distractors in Failing et al. (2015) possibly also captured attention covertly. As participants were explicitly told before the experiment what the high and low reward-value associated colors were, the distractors explicitly conveyed the informational value of signaling the reward magnitude that could be earned on a particular trial. Therefore it is possible that participants covertly oriented their attention towards the reward associated distractor stimuli. Along the same line of reasoning used in Le Pelley et al. (2015), associative reward learning in Failing et al. (2015) thus possibly involved superstitious instrumental conditioning. Here, by preventing covert shifts of spatial attention towards the reward signaling stimuli in the reward conditioning phase of Experiment 2, the present results unequivocally provide evidence that value-driven attentional capture can occur as a result of pure Pavlovian associative reward learning. This is crucial as it suggests that associative reward learning can be independent of training a covert or overt attentional orienting response towards the reward predictive stimuli, which implies that associative learning of the signal value of reward associated stimuli underlies value driven attentional capture.

Given that associative reward learning was completely independent of the assigned task, the results suggest that learning stimulus-reward contingencies from the environment can occur rather automatically. In a recent study, in which assessing the effect of value-driven attentional capture was interwoven with associative reward learning, Pearson et al. (2015) demonstrated that value-driven attentional capture was automatic and impenetrable for strategic attentional control. Participants were explicitly informed that looking at the distractor that signaled the magnitude of reward that could be earned on that trial resulted in an omission of that reward. The results showed that the explicit instructions could not minimize or counteract value-driven attentional capture, and the size of the attentional capture effect was not different for participants who did or did not receive explicit information about the stimulus-reward contingencies. In the present study, explicit information regarding the stimulus reward-contingencies were omitted on purpose, but, nevertheless, the results show that the color-reward contingencies were learned during the reward conditioning phase. In fact, during associative reward learning, participants were even instructed to ignore all stimuli, except for the fixation cross at the center of the screen. Thus, while participants were focused on whether the fixation cross would change, high and low rewards were automatically administered following the presentation of the reward signaling stimuli. Furthermore, to prevent coincidental instrumental learning, we ensured that the fixation cross never changed at the time the reward stimulus display or reward feedback was presented, thereby avoiding that the required response for a fixation change was mistakenly interpreted to result in reward delivery. This suggests that there was no incentive whatsoever for participants to pay attention to the reward signaling stimuli, as the reward signaling stimuli were completely task-irrelevant, and the reward structure was completely independent of any response. This is unique for the present work as in previous studies (Failing et al. 2015; Le Pelley et al., 2015; Mine & Saiki, 2015; Pearson et al., 2015) rewards were delivered only following a correct response. Although it is reasonable to assume that participants may have attended the reward-value associated stimuli in Experiment 2, as they were presented at fixation, the reward signaling stimuli never appeared simultaneously with a fixation change on which a response was required. Furthermore, the reward-value associated stimuli in Experiment 1 never appeared on a task-relevant spatial location, thereby providing absolutely no incentive to pay attention to them. Nevertheless, the results show that high compared to low reward-value associated distractors captured attention more strongly in the non-reward test phase that followed Pavlovian conditioning. This implies that the stimulus-reward contingencies were learned automatically during the reward conditioning phase of both experiments, despite the fact that the reward signaling stimuli served as temporally (and spatially) task-irrelevant distractors that had to be ignored. Thus, the present study shows that the value signal can be learned even if every incentive to pay attention to the reward structure is removed, while Pearson and colleagues showed that providing explicit information about the reward structure could not counteract the automatic effect of value-driven attentional capture. Together, these studies provide converging evidence that Pavlovian reward learning and value-driven attentional capture are automatic and occur independently of strategic attentional control.

To summarize, we show in two experiments that high compared to low reward-value associated stimuli capture attention more strongly in a non-reward test phase, after Pavlovian learning of stimulus-reward contingencies in a preceding reward conditioning phase. The present results suggest that value-driven attentional capture can occur following associative reward learning of temporally and spatially task-irrelevant distractors that signal the magnitude of available reward (Experiment 1) and that value-driven attentional capture is independent of training spatial shifts of attention towards the reward signaling stimuli (Experiment 2). The present study is the first to show associative reward learning through pure Pavlovian conditioning, as the reward signaling stimuli and reward administration were completely independent of the assigned task and any response during associative reward learning. This confirms and strengthens the idea that Pavlovian reward learning underlies value-driven attentional capture.
